# A Single *cis* Element Maintains Repression of the Key Developmental Regulator *Gata2*

**DOI:** 10.1371/journal.pgen.1001103

**Published:** 2010-09-09

**Authors:** Jonathan W. Snow, Jennifer J. Trowbridge, Tohru Fujiwara, Nikla E. Emambokus, Jeffrey A. Grass, Stuart H. Orkin, Emery H. Bresnick

**Affiliations:** 1 Division of Hematology-Oncology, Children's Hospital Boston, Boston, Massachusetts, United States of America; 2 Harvard Medical School, Boston, Massachusetts, United States of America; 3 Dana-Farber Cancer Institute, Boston, Massachusetts, United States of America; 4 Harvard Stem Cell Institute, Boston, Massachusetts, United States of America; 5 Wisconsin Institutes for Medical Research, University of Wisconsin School of Medicine and Public Health, Madison, Wisconsin, United States of America; 6 Howard Hughes Medical Institute, Boston, Massachusetts, United States of America; Medical Research Council Human Genetics Unit, United Kingdom

## Abstract

In development, lineage-restricted transcription factors simultaneously promote differentiation while repressing alternative fates. Molecular dissection of this process has been challenging as transcription factor loci are regulated by many *trans*-acting factors functioning through dispersed *cis* elements. It is not understood whether these elements function collectively to confer transcriptional regulation, or individually to control specific aspects of activation or repression, such as initiation versus maintenance. Here, we have analyzed *cis* element regulation of the critical hematopoietic factor *Gata2*, which is expressed in early precursors and repressed as GATA-1 levels rise during terminal differentiation. We engineered mice lacking a single *cis* element −1.8 kb upstream of the *Gata2* transcriptional start site. Although *Gata2* is normally repressed in late-stage erythroblasts, the −1.8 kb mutation unexpectedly resulted in reactivated *Gata2* transcription, blocked differentiation, and an aberrant lineage-specific gene expression pattern. Our findings demonstrate that the −1.8 kb site selectively maintains repression, confers a specific histone modification pattern and expels RNA Polymerase II from the locus. These studies reveal how an individual *cis* element establishes a normal developmental program via regulating specific steps in the mechanism by which a critical transcription factor is repressed.

## Author Summary

Different cell types are formed and maintained by proteins called transcription factors that directly bind to specific DNA sequences to activate or repress gene expression. While numerous DNA sequences bound by transcription factors are established, many questions remain unanswered regarding how they function at specific sites located at distinct chromosomal regions. As a model to study this process, we examined the regulation of a gene controlling red blood cell development, *Gata2*, by the transcription factor GATA1. In the DNA sequence upstream of *Gata2*, there are several sites that GATA1 is known to bind to; however, it is unclear whether these binding sites work together or independently to control expression of *Gata2*. To study this, we engineered mice to specifically remove one of these GATA1-binding sites. We found that removal of this single site reactivated expression of *Gata2* in a specific stage of red blood cell development where *Gata2* is normally not expressed, caused a block in differentiation of these cells, and changed the histone modification pattern specifically in the region upstream of *Gata2*. This work supports a model in which individual transcription factor binding sites within regions of multiple binding sites can independently and distinctly regulate gene expression during development.

## Introduction

Metazoan development is characterized by complex transcriptional programs specified by gene regulatory networks [1], [2]. Transcription factors in these networks occupy specific *cis* elements at target gene loci where they modulate chromatin remodeling and modification, and thereby transcription. The covalent modification of histones to yield specific histone marks promotes either the activation or repression of transcription [3]. Models of gene regulation have led to an attractive paradigm in which repression occurs in sequential stages of increasing stability [4]. While transcription factors bind and recruit chromatin-modifying and remodeling proteins, the relative contribution of individual *cis* elements residing within clusters of *cis* elements to the transcriptional control of endogenous loci is incompletely understood.

GATA factor cross-regulation represents an instructive model system for investigating the contribution of individual *cis* elements to the initiation and maintenance of transcriptional repression. The GATA family of transcription factors plays diverse roles in multiple developmental contexts [5]. GATA factors are often expressed in an overlapping but reciprocal pattern, such that expression of one GATA factor increases as expression of another decreases. For example, GATA-1 directly represses *Gata2* transcription via displacing GATA-2 from chromatin sites at its own locus, a process termed a “GATA Switch” [6], [7].

GATA factor function has been extensively studied in the context of hematopoiesis, where GATA-1, GATA-2, and GATA-3 are key regulators. GATA-2 has a broad role in hematopoietic development, as demonstrated by impaired hematopoiesis in *Gata2* knock-out mice resulting in lethality during midgestation [8], [9]. GATA-1 is critical for the production of red blood cells and platelets [10], and GATA-3 is required for specification of T cells [11]. Forced expression of GATA-2 blocks erythroid development [12], [13], [14], leading to a model in which GATA-1-mediated repression of *Gata2* through specific *cis* elements is required for differentiation. Genome-wide studies revealed GATA-1 occupancy at only a small subset of *cis* elements in the genome [15]. These *cis* elements exist as single or more complex GATA motifs, although the functionality of different permutations of GATA motifs at endogenous loci has not been investigated.

The role of individual GATA-binding sites in gene regulation has been investigated extensively at the *Gata2* locus, where several conserved GATA motif-containing regions span approximately 100 kb of the locus [16]. To test whether GATA switch sites function collectively or independently to regulate *Gata2* expression, and to investigate the underlying mechanisms, we generated mice lacking one of these regulatory regions residing −1.8 kb upstream of the *Gata2* promoter. We find that while this site is not essential for *Gata2* expression in hematopoietic progenitors or initiation of *Gata2* repression during erythropoiesis, it maintains *Gata2* repression in erythroblasts. Molecular analyses demonstrate that loss of the −1.8 kb site reduces GATA-1 binding, allows for increased RNA Polymerase II (Pol II) occupancy at the locus, and results in changes in select histone marks. Further, elimination of the −1.8 kb site dysregulates *Gata2* transcriptional control, disrupts the GATA-2-dependent genetic network, and interferes with red blood cell maturation. These results highlight the qualitatively distinct activities of individual *cis* elements in specific aspects of gene repression during development.

## Results

### Targeted deletion of the *Gata2* −1.8 kb *cis* element

**Figure 1:**
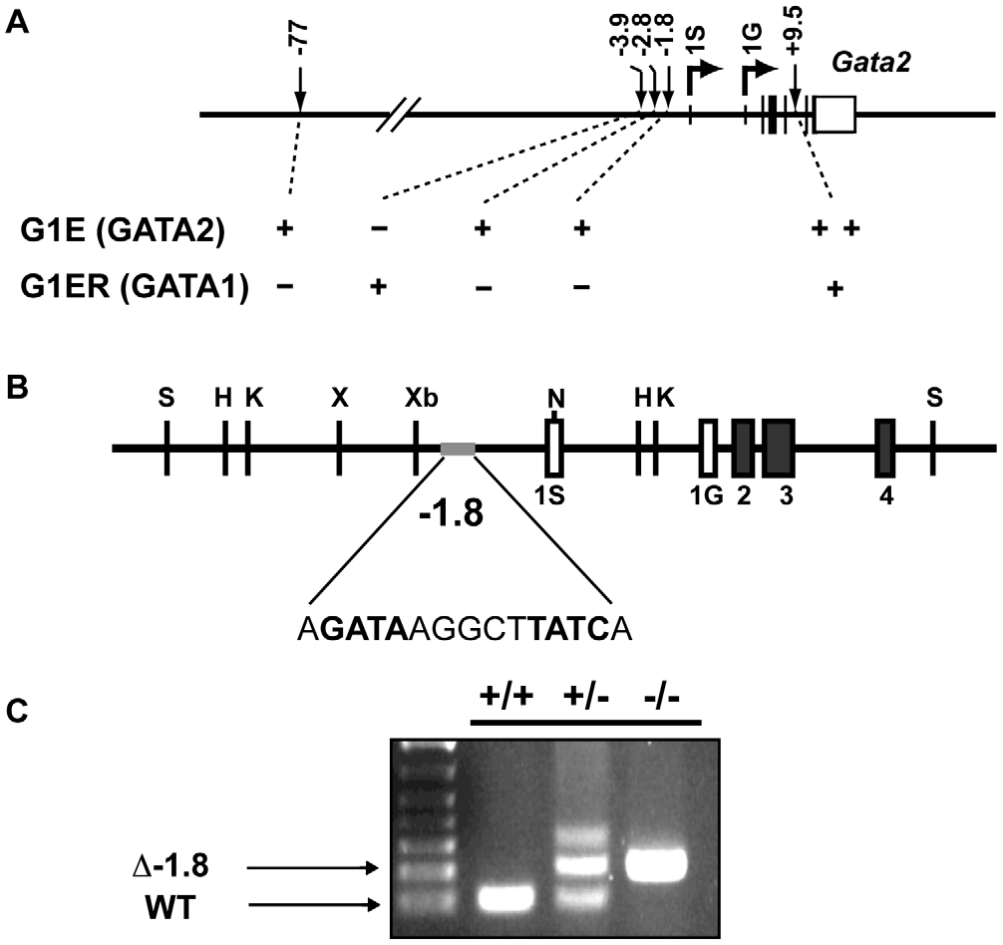
Generation of mice lacking the −1.8 kb site of the *Gata2* locus. Diagram of GATA binding sites involved in the regulation of hematopoietic expression of *Gata2* from its two alternate first exons, 1S and 1G, and their functional categories as defined by GATA-factor binding in G1E cells expressing only GATA2 and G1ER cells expressing only GATA1 (A). The region of the *Gata2* locus targeted in generation of mouse strain deficient for a palindromic GATA binding site 1.8 kb upstream of the *Gata2* transcriptional start site, referred to as the Δ-1.8 allele (B). PCR genotyping of mice deficient for the −1.8 GATA-binding site and littermate controls (C).

Previous studies in erythroid cell lines [17]–[22] and transgenic mouse models [23]–[25] have identified five GATA-binding regions upstream and in an intron of the *Gata2* locus (Figure 1A). It remains unknown whether these regions function collectively to confer *Gata2* transcriptional regulation, or if individual regions function uniquely at specific developmental stages and/or in select cell types. The site at −1.8 kb is of considerable interest, since it possesses strong GATA-2 binding activity that is lost upon repression [17]. Thus, we reasoned that removal of this site would phenocopy GATA-2-deficiency. As definitive analysis of *cis* element function requires genetic ablation of endogenous loci, we generated a mouse strain lacking the palindromic GATA-binding site 1.8 kb upstream of the *Gata2* transcriptional start site (Δ-1.8 allele) (Figures 1B, S1). Mice homozygous for the Δ-1.8 allele were born at expected Mendelian ratios, as assessed by PCR genotyping (Figure 1C.), implying that embryonic development was largely unaffected. Morphologically, E12.5 wild-type and mutant embryos were similar (Figure S2A), and adult mutant mice lacked gross abnormalities (data not shown).

**Figure 2:**
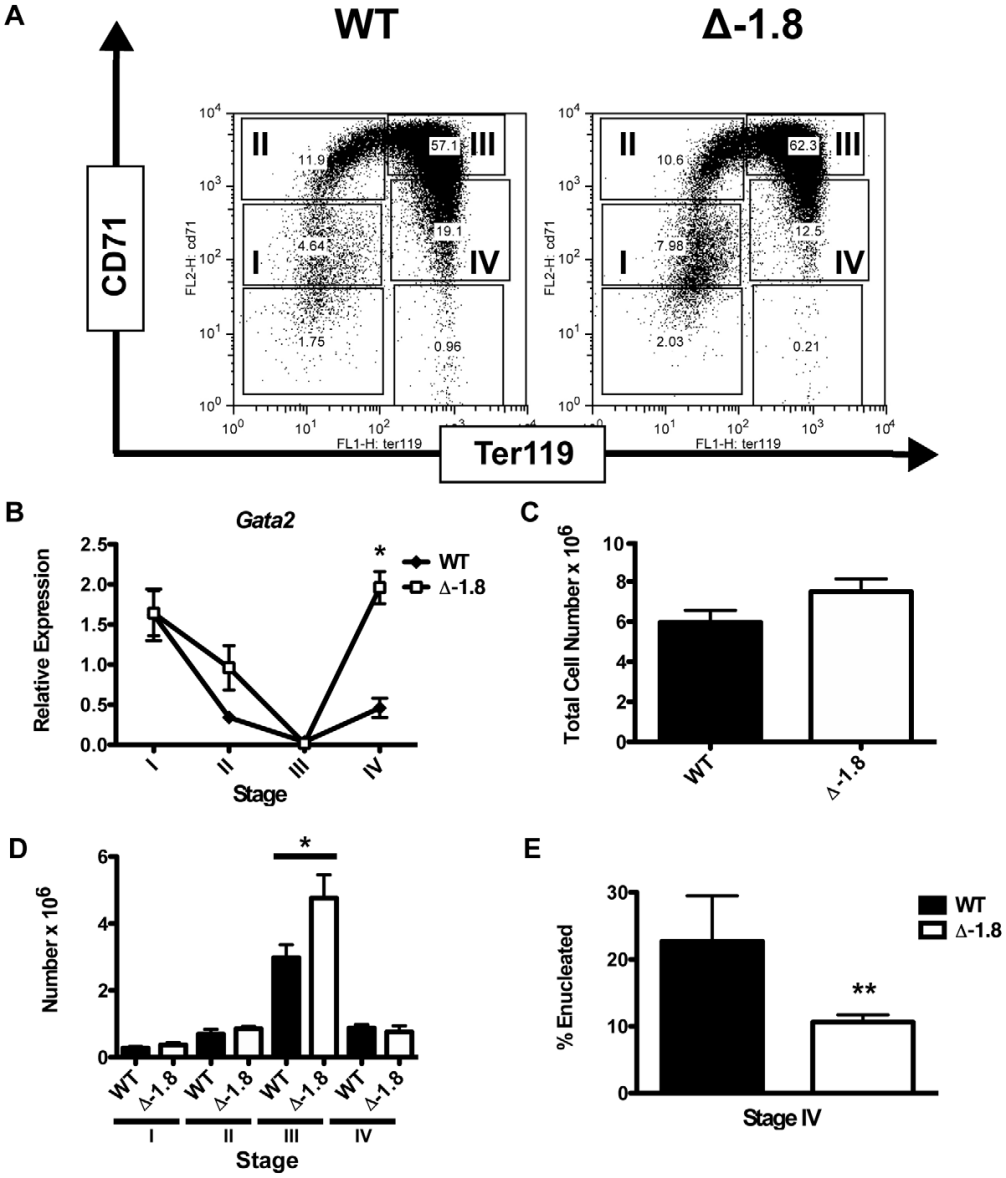
Alterations in *Gata2* expression in late erythroid development in Δ-1.8 mice. Representative FACS plots of E12.5 fetal liver cells stained for CD71 and Ter119 from wild-type and Δ-1.8 embryos (A). *Gata2* expression in Stage I through Stage IV sorted erythroid cells from wild-type and Δ-1.8 embryos normalized to β-actin expression (B). Total fetal liver cellularity in wild-type and Δ-1.8 embryos (C). Absolute number of cells in Stage I through Stage IV in fetal liver cells from wild-type (n = 4) and Δ-1.8 embryos (n = 3) (D). Percentage of Stage IV cells that are enucleated (negative for Draq5 staining) from wild-type (n = 8) and Δ-1.8 (n = 8) embryos (E).

We analyzed fetal liver erythropoiesis in Δ-1.8 mice for alterations in *Gata2* expression. Using fluorescence-activated cell sorting with the erythroid markers CD71 and Ter119 [26], we isolated cells from Stages I, II, III, and IV, corresponding to CD71^lo^Ter119^−^ (committed erythroid progenitors, Stage I), CD71^hi^Ter119^−^ (proerythroblasts, Stage II), CD71^hi^Ter119^+^ (basophilic erythroblasts, Stage III), and CD71^lo^Ter119^+^ (late erythroblasts, Stage IV) (Figure 2A). In wild-type mice, *Gata2* was most highly expressed in Stage I progenitors, after which it was repressed in Stages II, III, and IV (Figure 2B). *Gata2* expression was modestly increased in Stage IV, to about one fifth of that observed in Stage I. In Δ-1.8 mice, *Gata2* expression was normal in Stage I, and decreased normally in Stage II and III, indicating that the −1.8 kb site is not required for initiation of GATA-1-mediated repression. However, *Gata2* expression was significantly elevated in Stage IV cells from the Δ-1.8 versus wild-type mice (p≤0.05) (Figure 2B). Thus, the −1.8 kb site is selectively required to maintain *Gata2* repression in Stage IV erythroblasts.

### Impaired erythroid development in Δ-1.8 mutant mice

To determine if GATA-2 derepression has functional consequences in erythropoiesis, we analyzed erythroid cells in E12.5 fetal livers from wild-type and Δ-1.8 mice. Total cell numbers from wild-type and mutant fetal livers were similar (Figure 2C). Cytospins of peripheral blood and fetal liver cells from wild-type and mutant E12.5 embryos had similar appearance upon May-Gruenwald-Giemsa staining (Figure S2B). At this stage in development, most of the embryonic blood is comprised of primitive erythroid cells. However, some enucleated definitive cells were detected in both wild-type and Δ-1.8 embryos (Figure S2B). Hematopoietic colony assays from wild-type and Δ-1.8 E14.5 fetal livers revealed that the total number of colonies and lineage distribution of colony types (representing multipotential and lineage-restricted progenitors) were similar (Figure S2C). Examination of cells spanning different stages of erythroid development revealed no difference in the absolute number of Stage I, Stage II or Stage IV erythroid progenitors. However, the absolute number of Stage III erythroid progenitors was increased significantly (p≤0.05) in the Δ-1.8 mice (Figure 2D). These results demonstrate that at E12.5, Stage III progenitors from Δ-1.8 mice expand relative to both their precursors and progeny, implying a block in the Stage III to Stage IV transition. The increased number of Stage III progenitors is in accordance with other models of ineffective erythropoiesis, in which impairment of erythroid cell maturation is accompanied by a compensatory increase in earlier red blood cell precursors [27]. The timing of this block corresponds to the stage at which *Gata2* is reactivated (Figure 2B), indicating that *Gata2* dysregulation perturbs erythroid development. To examine this further, we utilized red blood cell enucleation as a cellular read-out of erythroid differentiation. Enucleation was measured using Draq5 to quantitate DNA content in Stage IV cells from wild-type and mutant embryos (a representative FACS plot is shown in Figure S2D). Stage IV cells in Δ-1.8 embryos contained a significantly reduced (>2-fold, p≤0.05) proportion of enucleated cells compared to those from wild-type, demonstrating that mutant cells fail to differentiate efficiently upon reactivation of *Gata2* expression (Figure 2E).

### *Gata2* reactivation in Δ-1.8 mice dysregulates GATA factor target genes

**Figure 3:**
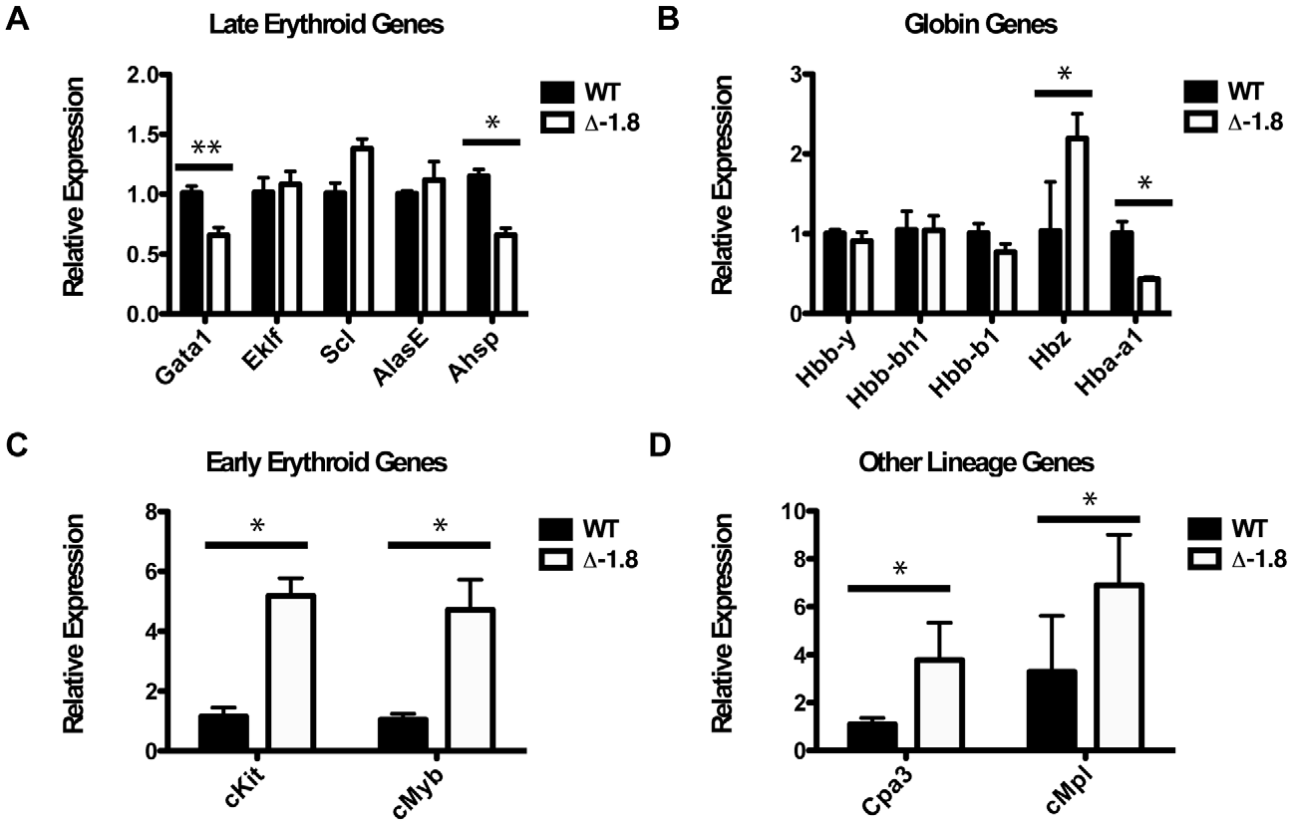
Disrupted gene expression in Δ-1.8 erythroblasts. Relative expression of genes in Stage IV sorted erythroid cells from wild-type and Δ-1.8 embryos; genes expressed in late erythropoiesis (A), globin genes (B), genes expressed in early erythropoiesis (C), and genes expressed in other lineages (D).

We reasoned that aberrant expression of GATA-2 target genes in Δ-1.8 mice might underlie the block in the transition from early to late erythroblasts. Increased *Gata2* expression could reactivate GATA-2 target genes expressed in early erythropoiesis, including those associated with proliferation, at a stage in which cells should exit the cell cycle. Alternatively, increased *Gata2* expression could aberrantly repress late erythroid genes necessary for efficient differentiation. Finally, abnormal reactivation of *Gata2* expression in cells expressing GATA-1 and other transcription factors involved in specifying alternate lineage programs could lead to the aberrant transcription of non-erythroid genes. To distinguish among these possibilities, we quantified gene expression in fetal liver erythroid cells from E12.5 mice. Several gene expression changes were apparent in Stage IV erythroblasts (Figure 3A). Expression of *Gata1* and *Eraf*, a globin chain stabilizing protein, were reduced by ∼40% (p≤0.01) and ∼50% (p≤0.05), respectively, in the late erythroblasts of Δ-1.8 versus wild-type mice. In contrast to *Gata1* and *Eraf*, most late erythroid genes examined, including the transcription factors *Scl*, *Eklf*, and the heme synthesis enzyme *Alas2*, were expressed at similar levels, indicating that erythroid genes are differentially sensitive to *Gata2* reactivation. Whereas expression of β-like globin genes (*Hbb-y*, *Hbb-bh1*, *Hbb-b1*) was normal (Figure 3B), expression of α-globin (*Hba-a1*) was reduced by 50% (p≤0.05) and ζ-globin (*Hba-x*) was increased by 2-fold (p≤0.05) (Figure 3B). We also examined two genes expressed early in erythropoiesis. Both *cMyb* and the established GATA-2 target *cKit* were upregulated 4-fold (p≤0.05) (Figure 3C). In mast cells and megakaryocytes, GATA-2 is expressed in combination with other transcription factors including SCL and GATA-1. As GATA-2 is aberrantly coexpressed with these factors in the Δ-1.8 erythroblasts, we examined select GATA-2 target genes from the mast cell and megakaryocyte lineages in our wild-type and mutant erythroblasts. *Cpa3*, active in mast cells, and *cMpl*, expressed in megakaryocytes, were upregulated 4- and 2-fold, respectively, in mutant versus wild-type erythroblasts (p≤0.05) (Figure 3D). These results indicate that *Gata2* reactivation is coupled with aberrant GATA-2 target gene expression. Given the dysregulation of genes associated with early progenitor proliferation, erythroid maturation, and alternate lineage fate, it is likely that these factors contribute in aggregate to the block in erythroid development.

### Defective stress erythropoiesis in Δ-1.8 mice

**Figure 4:**
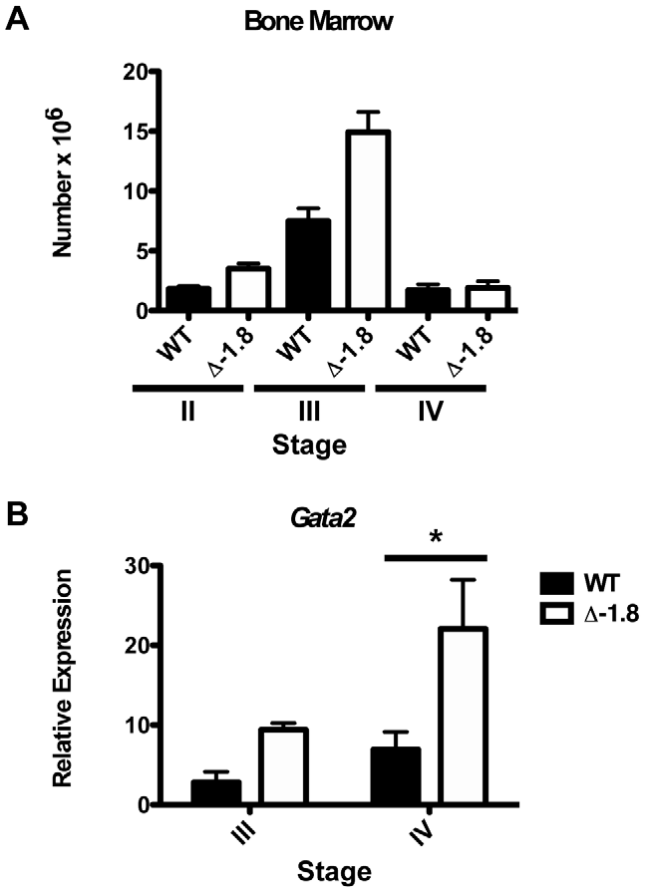
*Gata2* dysregulation during stress erythropoiesis in Δ-1.8 mice. Total number of erythroid progenitors in the bone marrow of wild-type (n = 4) and Δ-1.8 (n = 6) mice 4 days post-phenylhydrazine injection (A). *Gata2* expression in Stage III and Stage IV erythroid cells sorted from the bone marrow of phenylhydrazine-treated wild-type and Δ-1.8 mice (B).

In contrast to the E12.5 fetal liver, erythroid progenitors isolated from the bone marrow of adult wild-type and Δ-1.8 mutant mice (Stage II–IV) did not reveal differences in *Gata2* expression (data not shown), indicating that *Gata2* transcription is differentially regulated during fetal and adult erythropoiesis. Adult erythropoiesis has several unique attributes relative to the fetal process, including differences in proliferative capacity and rate of transit through the differentiation program [28], [29]. Such differences might explain the ontogenic specificity of *Gata2* reactivation. We reasoned that stress erythropoiesis in the adult, which resembles fetal liver erythropoiesis [28]–[32], might shift the regulation of *Gata2* expression to a state mimicking that in the fetus. To establish stress erythropoietic conditions, peripheral anemia was induced through phenylhydrazine-mediated red blood cell lysis. Examination of erythropoietic recovery in Δ-1.8 mice revealed no differences in hematocrit, implying that there is no deficiency in recovery from acute anemia in these mice (data not shown). However, analysis of erythropoietic progenitor production in the bone marrow during recovery revealed that the absolute number of Stage III erythroid progenitors was significantly increased in Δ-1.8 mice (p≤0.05) (Figure 4A), while the number of Stage IV erythroid progenitors was similar, again indicating a block in the transition from Stage III to Stage IV. The increased number of Stage III cells is likely an indirect effect due to the increased sensitivity of Δ-1.8 mice to stress-induced ineffective erythropoiesis [27]. Expression analysis of sorted populations from the bone marrow of these mice showed that *Gata2* transcription is increased significantly (p≤0.05) in Stage IV cells from Δ-1.8 mice (Figure 4B). These results mimic those obtained with E12.5 fetal liver (Figure 2B,D), indicating that the −1.8 kb site controls *Gata2* expression in both stress and fetal erythropoiesis.

### Δ-1.8 cells possess altered nucleoprotein architecture of the *Gata2* locus

**Figure 5:**
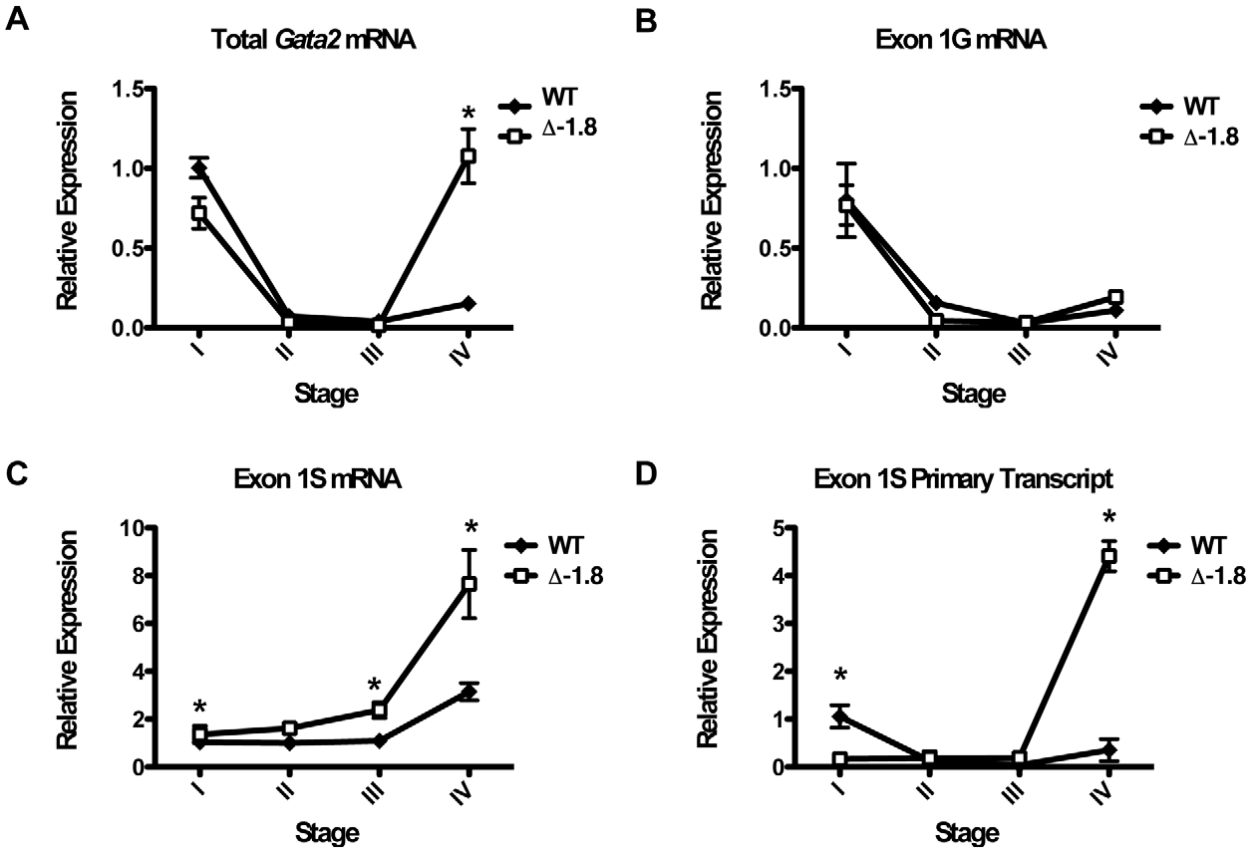
Promoter usage in Δ-1.8 *Gata2* dysregulation. Total *Gata2* mRNA (A), mRNA selectively arising from the 1G (B) or the 1S (C) promoter, and primary, unspliced transcript arising from the 1S promoter (D) in Stage I through Stage IV sorted erythroid cells from wild-type and Δ-1.8 embryos at E14.5.

*Gata2* is transcribed from two alternate promoters, termed 1S and 1G, leading to two transcripts with different first exons [33]. To determine whether the loss of *Gata2* repression in Δ-1.8 erythroid cells (Figure 5A) reflects increased transcripts derived from one or both of the promoters, we used primers specific for mature forms of the 1S and 1G transcripts. The majority of *Gata2* transcripts expressed in Stage I were derived from the 1G promoter and were repressed in Stage II–IV similarly in wild-type and Δ-1.8 cells (Figure 5B). mRNA expression from the 1S promoter was increased nearly 8-fold (p≤0.05) in Δ-1.8 Stage IV cells relative to Stage I cells. While wild-type cells exhibited increased 1S-derived mRNA at Stage IV relative to Stage I, this increase was significantly smaller (Figure 5C). Quantitation of primary, unspliced transcripts derived from the 1S promoter revealed an even more striking increase in 1S-derived transcript from Δ-1.8 Stage IV cells (∼10-fold relative to Stage I) compared to wild-type cells from the same stage, which did not demonstrate any appreciable increase (p≤0.05) (Figure 5D). Together, these results demonstrate that loss of the −1.8 kb site selectively reactivates transcription from the 1S promoter in erythroid cells.

**Figure 6:**
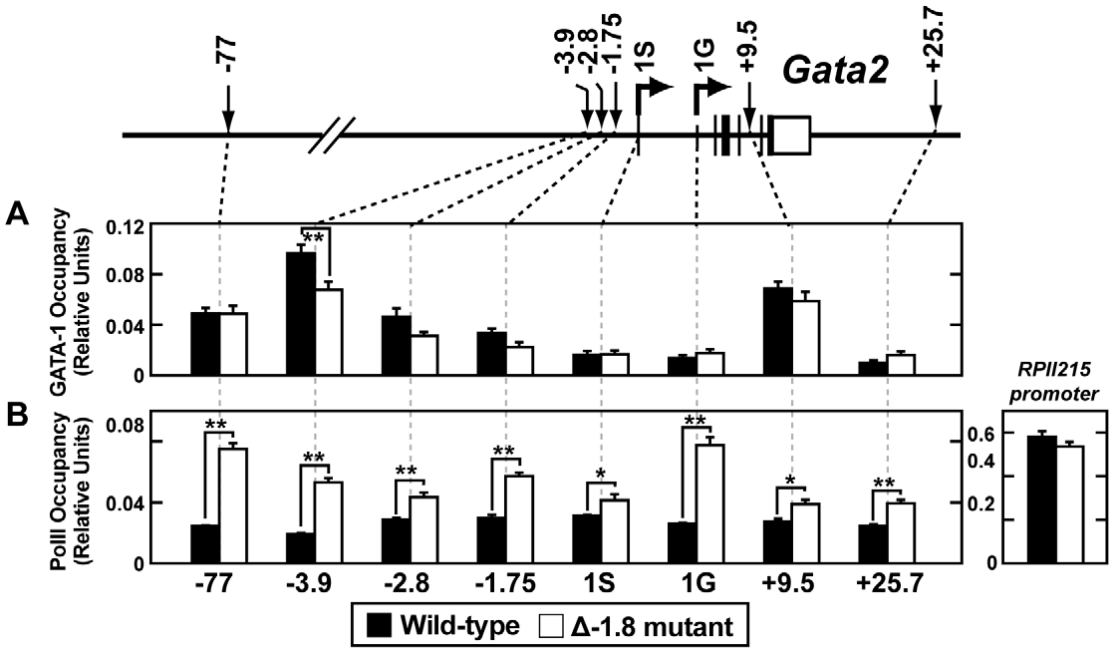
Loss of the −1.8 kb site leads to increased RNA Pol II occupancy of the *Gata2* locus. Quantitative ChIP analysis across the *Gata2* locus using antibodies to GATA-1 (A) and RNA Pol II (B) in whole fetal liver cells from wild-type and Δ-1.8 embryos at E14.5. Calculations were derived using percentage of input and were normalized using relative units which were determined by defining 9% input sample as 1.0.

As expected, quantitative chromatin immunoprecipitation (ChIP) analysis of E14.5 fetal liver cells demonstrated reduction of GATA-1 occupancy at the −1.75 kb (used as a surrogate for measuring occupancy at the deleted −1.8 kb site) and the −2.8 kb sites (p = 0.057 and 0.058 respectively) and the proximal GATA-binding regions at −3.9 kb, (p≤0.01); occupancy was not significantly altered at the distal −77 and +9.5 kb sites (Figure 6A). ChIP analysis of Pol II demonstrated significantly increased occupancy at all sites examined upon mutation of the −1.8 kb site, with notable increases at the −77 kb enhancer (p≤0.01), the −1.75 kb site (p≤0.01) and the 1G promoter (p≤0.01) (Figure 6B). Importantly, Pol II occupancy of a distant gene (*RPII215*) did not change upon loss of the −1.8 kb site (Figure 6B), providing evidence for locus specificity. ChIP analysis of GATA-2 occupancy yielded signals near background levels, consistent with GATA-2 expression being below the limit of detection in this assay (data not shown). Average preimmune values for the wild-type and Δ-1.8 cells were 0.0018±0.00027 and 0.0041±0.0017, respectively.

**Figure 7:**
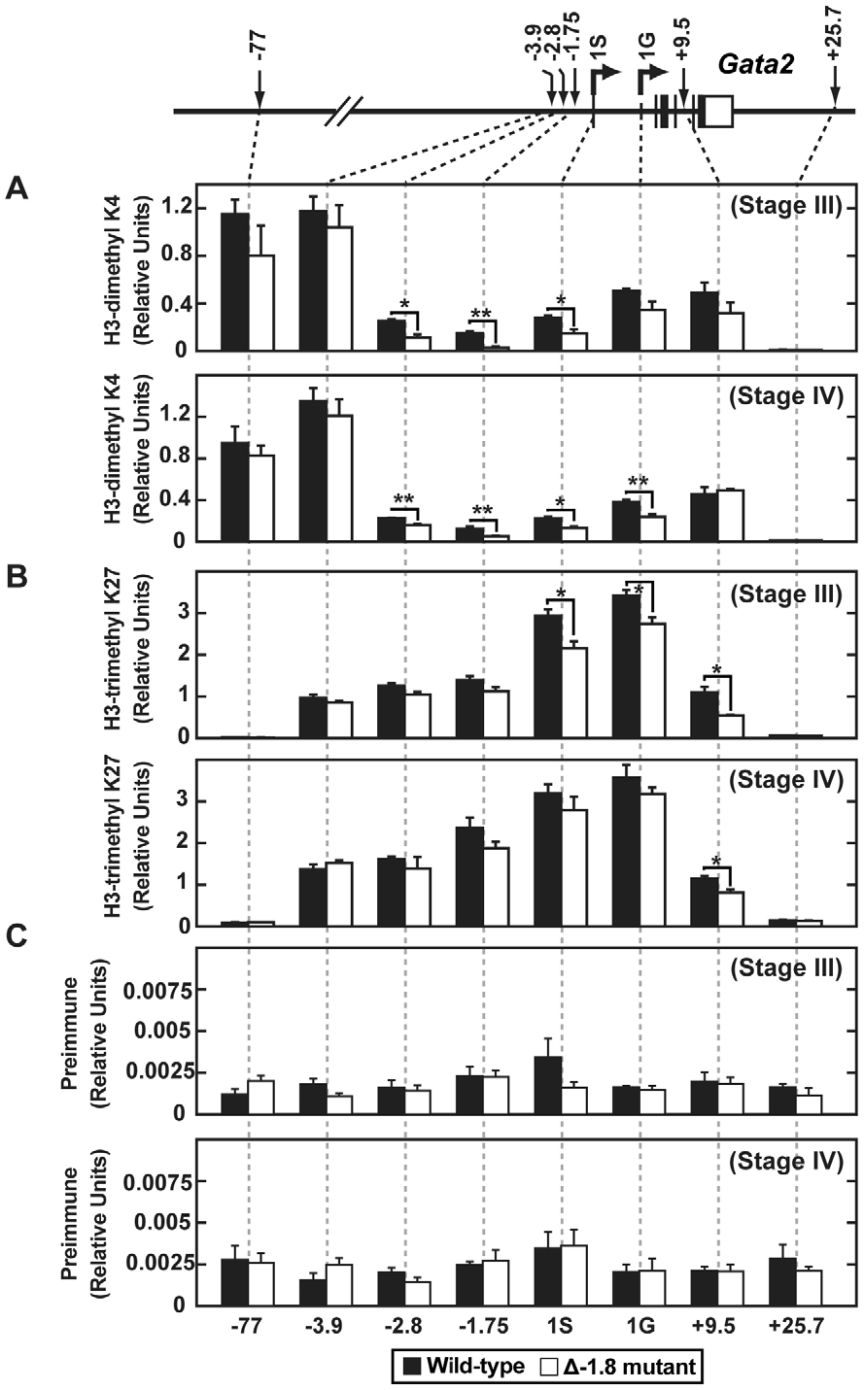
Loss of the −1.8 kb site alters dimeH3K4 and trimeH3K27 marks in Stage III and IV erythroblasts. Quantitative ChIP analysis across the *Gata2* locus using antibodies to dimeH3K4 (A) and trimeH3K27 (B) and preimmune (C) in Stage III and Stage IV cells sorted from wild-type and Δ-1.8 embryos at E14.5. Calculations were derived using percentage of input and were normalized using relative units which were determined by defining 9% input sample as 1.0.

To analyze histone modifications within the erythroid populations in which we observed an altered phenotype on removal of the −1.8 kb site (Figure 2), we performed quantitative ChIP on sorted fetal liver Stage III and IV cells. We quantitated dimeH3K4 and trimeH3K27, two marks shown to be associated with repression at the *Gata2* locus [17], [21], [34]. dimeH3K4 was significantly reduced at the −1.75 kb site, neighboring proximal regulatory regions, and the 1S promoter in both Stage III and Stage IV Δ-1.8 cells (p≤0.05) (Figure 7A). The repressive mark trimeH3K27 was decreased to a small extent at the promoters in Stage III Δ-1.8 cells (p≤0.05) (Figure 7B). Preimmune values were similar between wild-type and −1.8 samples (Figure 7C). These results in primary erythroid progenitors provide direct evidence that the −1.8 kb *cis* element contributes to the maintenance of the dimeH3K4 mark in erythroid cells.

Contribution of the dimethylH3K4 modification to transcriptional regulation is incompletely understood [35]–[37]. By contrast, the trimethylH3K4 mark is thought to play a critical role in promoting gene activation [38], [39]. Also, recent attention has been focused on the monomethylH3K4 mark as an important regulator of enhancer elements [38], [40]. We reasoned that loss of dimethylH3K4 might play an indirect role by providing a substrate for increases in the mono- or trimethyl forms of H3K4. However, ChIP using E14.5 whole fetal liver cells revealed that the levels of trimeH3K4 were unchanged at all sites examined (Figure S3A). Even more strikingly, the levels of monomeH3K4 were reduced at the −2.8 (p≤0.05) and −1.75 kb sites (p≤0.01), as well as the 1G promoter (p≤0.05) (Figure S3B), similar to the reduction in dimeH3K4 observed in whole fetal liver cells (data not shown) and in sorted cells (Figure 7). Total H3 and preimmune values for ChIP using whole fetal liver cells were similar between wild-type and Δ-1.8 samples (data not shown).

These data indicate that loss of GATA-1 binding at the deleted −1.8 kb *cis* element leads to decreased GATA-1 occupancy at sites up to several kilobases away, reductions in dimeH3K4 and monomeH4K4 marks in the regulatory regions, and increased RNA Pol II occupancy. We propose a model in which this altered nucleoprotein structure favors a transcriptionally active locus, thereby permitting *Gata2* reactivation.

### CpG island methylation of the *Gata2* 1S promoter is independent of the −1.8 site

**Figure 8:**
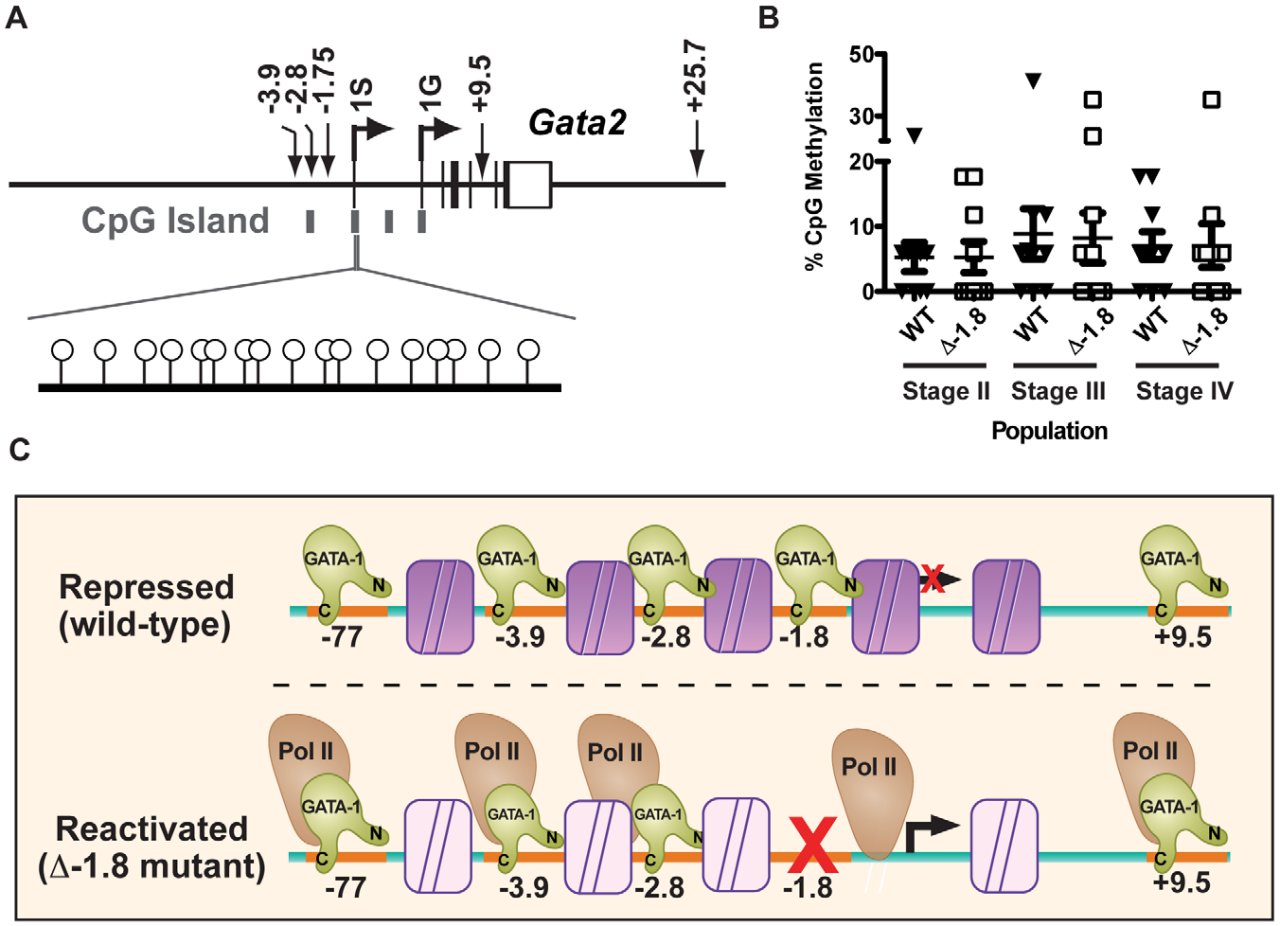
*Gata2* promoter methylation does not require the −1.8 kb site. CpG island locations within the *Gata2* locus (A). Percentage of CpG methylation within the 3′ region of the 1S promoter in Stage II, III and IV erythroid cells from E14.5 wild-type and Δ-1.8 fetal liver (B). A model of the maintenance of *Gata2* locus repression, mediated through maximal GATA-1 binding, repressive histone marks (purple), and complete Pol II expulsion. In the absence of the −1.8 kb site, GATA-1 binding is diminished, leading to a reduction in repressive histone marks (transparent) and allowing Pol II occupancy (C).

The *Gata2* locus contains four CpG islands [17] located at the −2.8 kb GATA-binding region, both the 1S and 1G promoters, and an unclassified region between these promoters (Figure 8A). Stable repression at loci characterized by CpG-rich promoters is thought to depend, in part, upon methylation of these promoters [4], [41]. In addition, tissue specific gene silencing of *Gata2* has been correlated with promoter methylation in some tissues [42], [43]. Thus, we tested whether methylation of the 1S promoter is important for stable repression in erythroid cells and whether the −1.8 kb *cis* element maintains repression through such a mechanism. Bisulfite sequencing was utilized to quantitate promoter methylation of a 3′ section of the 1S CpG island within sorted populations of Stage II–IV erythroid progenitors from wild-type and mutant mice. In wild-type mice, the CpG island located at the *Gata2* 1S promoter was largely unmethylated in Stage II, Stage III, and Stage IV progenitors, with an average methylation of 5.2%, 8.9%, and 7.1%, respectively (Figure 8B). As no specific residues were hypermethylated (Figure S3C), these data imply that methylation of the 1S CpG island is not important for maintenance of repression in these cells. In Δ-1.8 mice, the 1S CpG island displayed similar levels of methylation in Stage II, Stage III, and Stage IV progenitors (5.9%, 8.2%, and 7.1%, respectively, Figure 8B). Thus, the stable repression of *Gata2* does not require DNA methylation of the 1S CpG island, and the −1.8 kb site maintains repression in Stage IV erythroblasts through other mechanisms including regulation of transcription factor occupancy, histone modifications, and Pol II access.

## Discussion

We have described a loss of function strategy in mice to establish definitively whether one of the *cis* elements previously implicated in the control of *Gata2* [17], [19], [21] functions independently or collectively with other *cis* elements to regulate *Gata2* transcription *in vivo*. Unexpectedly, the endogenous −1.8 kb site is dispensable for activation of *Gata2* and the initiation of repression, but instead selectively maintains *Gata2* repression in terminally differentiating cells. Deletion of the −1.8 kb site reactivates *Gata2* expression resulting in an erythroid maturation block, likely due to improper regulation of the reciprocally controlled *Gata1*, genes involved in globin synthesis, genes expressed earlier in erythropoiesis, and genes associated with other hematopoietic lineages.

While one or more additional GATA-binding sites in the locus must contribute to the initiation of repression, we propose that maintenance of repression is mediated through GATA-1 binding at the −1.8 kb site of the *Gata2* 1S promoter. In a wild-type setting, transcription factor binding and histone modifications lead to Pol II expulsion in a locus-wide manner to establish stable repression (Figure 8C). In the absence of the −1.8 kb *cis* element, GATA-1 occupancy is lost at this site. Our results demonstrate that locus-wide Pol II expulsion requires maximal GATA-binding at the 5′ proximal regulatory regions, highlighting a critical role for the −1.8 kb site in regulating Pol II occupancy. The loss of GATA-1 occupancy in the absence of the −1.8 kb site results in a reduction in one of the marks associated with repression at this locus, dimeH3K4, while having minimal effects on another repressive mark, trimeH3K27. Intriguingly, dimeH3K4 decreases in a manner consistent with expectations from studies in cultured cells [17], [21]. While this mark is commonly associated with activation in most contexts, recent genome-wide analysis studies have implicated this mark in both activation and repression [38], [39], and therefore our understanding of the functional consequences of this mark seems incomplete [35]–[37]. Two possibilities may account for the similarity of the dimethylH3K4 level in Δ-1.8 cells between repressed (III) and reactivated (IV) stages. First, dimethylH3K4 may not be the critical modification mediating maintenance of repression. Alternatively, other stage-specific factors in the nuclear milieu may lead to differential sensitivities to dimethylH3K4 between the repressed (III) and reactivated (IV) stages.

Substantial reduction in both dimeH3K4 and monomethylH3K4 were observed upon loss of the −1.8 kb site without a concomitant increase in trimethylH3K4. These findings suggest that the methylation states of H3K4 are regulated independently and locally through complexes recruited to the −1.8 kb GATA-binding site. These observations are in accordance with the finding that dimeH3K4 positive, trimethylH3K4 negative, marks are present at a subset of developmentally regulated hematopoietic genes [44]. Thus, our data highlight a potential role for these H3K4 marks in regulating transcription. It is interesting to note that the trimethylK27 mark, associated with GATA-1-mediated repression of the *Gata2* locus [34], is not affected by the −1.8 kb GATA-binding site. In addition, reduction of H3K27 trimethylation, widely accepted as a repressive mark [45], does not appear to be required to reactivate gene expression at the *Gata2* locus, perhaps indicating that it is involved selectively in the initiation of repression. Recent genome-wide analysis has also shown that H3K27 methylation is not merely present or absent, but rather increases quantitatively as the activity of the gene decreases [38], [40], suggesting that the level of transcriptional reactivation observed is within the range allowed by the H3K27 methylation level at this locus. Finally, in many cases, CpG rich promoters require hypermethylation of associated CpG islands for stable repression [4], [37]. We find that the CpG island at the *Gata2* 1S promoter lacks high levels of methylation during stable repression, and that loss of the −1.8 site does not affect methylation levels. This data further supports a model in which −1.8 kb site-dependent histone marks maintain stable repression.

We propose therefore that loss of GATA-1 binding and key repressive marks, including dimethyl- and monomethyl-H3K4, result in a locus permissive for Pol II occupancy and reactivation of transcription. This model predicts that a specific protein or proteins are recruited by GATA-1 to the −1.8 kb site to maintain repressive chromatin structure. GATA-1 is known to interact with CBP [46], HDACs 1 and 2 [47], [48], LSD1 [49], BRG1 [50], and polycomb repressive complex 2 (PRC2) [34]. As no GATA-1-interacting proteins have been reported that possess the requisite methyltransferase activity to establish the dimeH3K4 histone mark that is lost in the −1.8 kb mutant, novel GATA-1-containing complexes may be required to maintain the −1.8 kb site-dependent histone marks. Ongoing genetic ablation studies examining the contribution of the other known GATA-binding regions to *Gata2* regulation and local chromatin architecture will be important for understanding the control of this complex locus.

Studies in multiple systems have led to a model of sequential gene repression during development [4], separable into distinct phases. Reversible repression is replaced by epigenetic mechanisms that alter the chromatin structure at the locus though modifications of histones, and in some cases DNA, to maintain stable repression. The results described herein support such a model and characterize molecular mechanisms associated with the selective maintenance of repression of an endogenous target gene by an individual *cis* element to confer normal developmental control.

## Materials and Methods

### Ethics statement

All animals were handled in strict accordance with good animal practice as defined by the relevant national and/or local animal welfare bodies, and the appropriate committee approved all animal work.

### Generation of mice containing *Gata2* Δ-1.8 knock-in allele

Briefly, to generate the −1.8 kb knock-in allele, we replaced the palindromic GATA sites (AGATAAGGCTTATCA) with two SalI sites in order to clone in a Neo resistance cassette flanked by loxP sites. Once the neo cassette is removed, the locus contains a single loxP site flanked by SalI sites. The new sequence does not contain any binding motifs known to be involved in hematopoietic development. In more detail, we first inserted a HpaI site into pBSK between NotI and SacI with an oligo. Then, we cloned a −7.2 kb to intron 1S fragment of the *Gata2* locus into pBSK with KpnI and HpaI. We then replaced the two palindromic WGATAR sites with a Sal I site via PCR and replaced the wild-type XbaI to NdeI fragment with this mutated version. Then, we cloned HSV-TK cloned into the SacII site of pBSK. Following this, we cloned a second SalI site into the XbaI site of pflox21 with an oligo and used the flanking SalI sites to clone this loxP-PGKneo-loxP cassette into the SalI site of the *Gata2*-containing pBSK (Figure S1A). We screened targeted CJ7 ES clones by PCR and confirmed correct targeting by Southern blotting. We used standard blastocyst injection techniques to generate chimeric mice and screened F1 pups for germline transmission using Southern blotting (Figure S1B,C). In some mice, the loxP-neomycin resistance gene was deleted by crossing with Gata1-Cre mice, which were of CD1/Swiss-Webster background. We confirmed Cre-mediated excision of neo from these mice using PCR and all further genotyping was performed by PCR (Figure 1C). Mice were backcrossed onto a C57/Bl6 background for a minimum of 6 generations and were housed in a specific pathogen-free animal facility.

### Fetal liver and bone marrow sampling

Fetal liver cells were obtained from embryos at E12.5 and E14.5 after timed matings. Mouse bone marrow cells were obtained from 8- to 12-week-old animals by crushing femurs and tibias with either Iscove modified Dulbecco medium (IMDM) or Phosphate Buffered Saline (PBS) supplemented with 2% fetal calf serum (Mediatech, Herndon, VA). Single cell suspensions of fetal livers and spleens were made by passage through 70 micron nylon mesh (Sefar America, Kansas City, MO) in PBS supplemented 2% fetal calf serum (Mediatech, Herndon, VA). Cells were kept on ice until use and counts were performed using a Beckman Coulter AcT10 hematological analyzer.

### Real-time reverse-transcriptase PCR

RNA was prepared from the described populations using the Trizol Kit (Invitrogen, San Diego, CA), DNAseI treated by RQ1 RNase-Free DNase (Promega, Madison, WI) and quantified. cDNA was synthesized using 1 µg of RNA with the iScript cDNA Synthesis Kit (Biorad, Hercules, CA). Typically, 1 µl of cDNA was then used as a template for quantitative PCR using the iQ SYBR Green Supermix (Biorad, Hercules, CA) in an iCycler thermocycler (Biorad, Hercules, CA). Primer sequences can be found in Text S1. Triplicate data sets were generated and results were normalized to β-actin reactions run in parallel.

### Complete blood count

Whole PB was analyzed on a Beckman Coulter AcT10 hematological analyzer. White blood cell and progenitor subsets were analyzed from peripheral blood by staining with Gr-1 and Mac1 or CD3 and B220 after red blood cell lysis using ACK (NH_4_Cl) lysis buffer.

### Flow-cytometric analysis and cell sorting

All antibodies for FACS were obtained from Pharmingen (San Diego, CA) or eBiosciences (San Diego, CA), and the following clones were used; Ly-76 (Ter-119), CD71 (C2), CD117 (2B8). Antibodies to surface markers of interest were used at 1∶60 dilution and after 30–60 minutes unbound antibody was washed away. In the case of biotinylated antibodies, streptavidin conjugated to various fluorochromes was added for the last 15–30 minutes of antibody incubation at 1∶100 dilution. For cell sorting experiments of erythroid progenitor subsets, fetal liver cells were stained with antibodies to CD71 and Ter119, and 7AAD was added to allow for exclusion of dead cells during sorting. For examination of enucleation, cells were stained with CD71 and Ter119 as above and incubated with Draq5 (Biostatus Limited, Leicestershire,United Kingdom) as per manufacturers instructions before analysis.

### Quantitative chromatin immunoprecipitation (ChIP) assay

Rabbit anti-GATA-1 and anti-GATA-2 polyclonal antibodies have been described previously [16], [21], [51]. Rabbit anti-Pol II (N-20, sc-899) was from Santa Cruz Biotech. Rabbit anti-acetyl-histone H3 (#06-599), anti-trimethyl-histone H3 (Lys 9) (#07-442), anti-trimethyl-histone H3 (Lys 27) (#07-449) and anti-dimethyl-histone H3 (Lys 4) (#07-030) were from Millipore. Real-time-PCR-based quantitative chromatin immunoprecipitation (ChIP) analysis was conducted as described [52]. Single-cell suspensions were isolated from E14.5 wild-type and Δ-1.8 fetal liver cells, respectively, and crosslinked with 1% formaldehyde. Samples were analyzed by real-time PCR (ABI Prism 7000) using primers designed by PrimerExpress™ 1.0 software (PE Applied Biosystems) to amplify regions of 75–150 bp that overlap with the appropriate motif. Product was measured by SYBR Green fluorescence in 20 µl reactions, and the amount of product was determined relative to a standard curve generated from titration of input chromatin. Calculations were derived using percentage of input and were normalized using relative units which were determined by defining 9% input sample as 1.0. Analysis of dissociation-curves post-amplification showed that primer pairs generated single products.

### Bisulfite sequencing

Bisulfite treatment of genomic DNA was performed as previously described using the Qiagen EpiTect Bisulfite Kit as per the manufacter's instructions. Sequence-specific PCR of the bisulfite-treated DNA was performed using primers specific to the murine *Gata2* 1S promoter (outside primers: F, 5′- TTGTGTGGTGAGGGTGTAG-3′, R, 5′- CAAATTTCTTTCCCTATTTTCT-3′; inside primers: F, 5′- TAGGTGGGGGAGAGTGTAG -3′, R, 5′- CAAATTTCTTTCCCTATTTTCT -3′. The PCR fragments were sub-cloned into the pCR®2.1-TOPO® vector (Invitrogen) and transformed into DH5α *E. coli* cells. Miniprep plasmid DNA was verified by EcoRI digestion and positive clones were sequenced using M13 forward (−20) or reverse primers.

### Statistical analysis

Data are presented as mean ± SEM. Statistical significance was assessed by two-sided Student's t-test.

## Supporting Information

Figure S1 Δ-1.8 targeting construct generation. Graphical representation of the generation of the targeting construct to replace the palindromic GATA-binding site −1.8 kb upstream of the 1S transcriptional start site with a loxP-flanked PGK-neomycin cassette (A). Southern blot strategy outlining the HindIII/SalI digested fragment sizes for the wild-type and targeted alleles, and probe hybridization sites (B). Southern blot of Δ-1.8 germline mice and wild-type (WT) littermates from tail tip genomic DNA (C).
(0.27 MB PDF)

Figure S2 E12.5 hematopoiesis in Δ-1.8 mice. Representative E12.5 wildtype (WT) and Δ-1.8 embryos (A). Cytospins of embryonic peripheral blood and fetal liver cells from E12.5 WT and Δ-1.8 embryos (B). Number of CFU-GEMM, CFU-GM, BFU-E, and CFU-E colonies per 10^4^ wild-type and Δ-1.8 E12.5 fetal liver cells (C). FACS histograms showing the proportion of enucleated cells from Stage IV erythroblasts within representative wild-type and Δ-1.8 fetal livers (D).
(0.99 MB PDF)

Figure S3 Loss of the −1.8 kb site leads to altered nucleoprotein architecture of the *Gata2* locus. Quantitative ChIP analysis across the *Gata2* locus using antibodies to trimethylH3K4 (A) and monomethylH3K4 (B) in whole fetal liver cells from wild-type and Δ-1.8 embryos at E14.5. Calculations were derived as above. Bisulfite sequencing of the 3′ region of the *Gata2* 1S promoter CpG island in WT and Δ-1.8 Stage II, Stage III, and Stage IV erythroid progenitors. Each line represents an individual sequenced clone; white circles denote unmethylated CpG dinucleotides, black circles denote methylated CpGs (C).
(0.49 MB PDF)

Text S1 Supporting Materials and Methods.
(0.05 MB DOC)
