# Supplementary figures and images for: A Single cis Element Maintains Repression of the Key Developmental Regulator Gata2
Source: PLoS Genet. 2010 Sep 9;6(9):e1001103. doi: 10.1371/journal.pgen.1001103 (PMC2936534; doi:10.1371/journal.pgen.1001103)

**A**

**$\Delta$ -1.8**

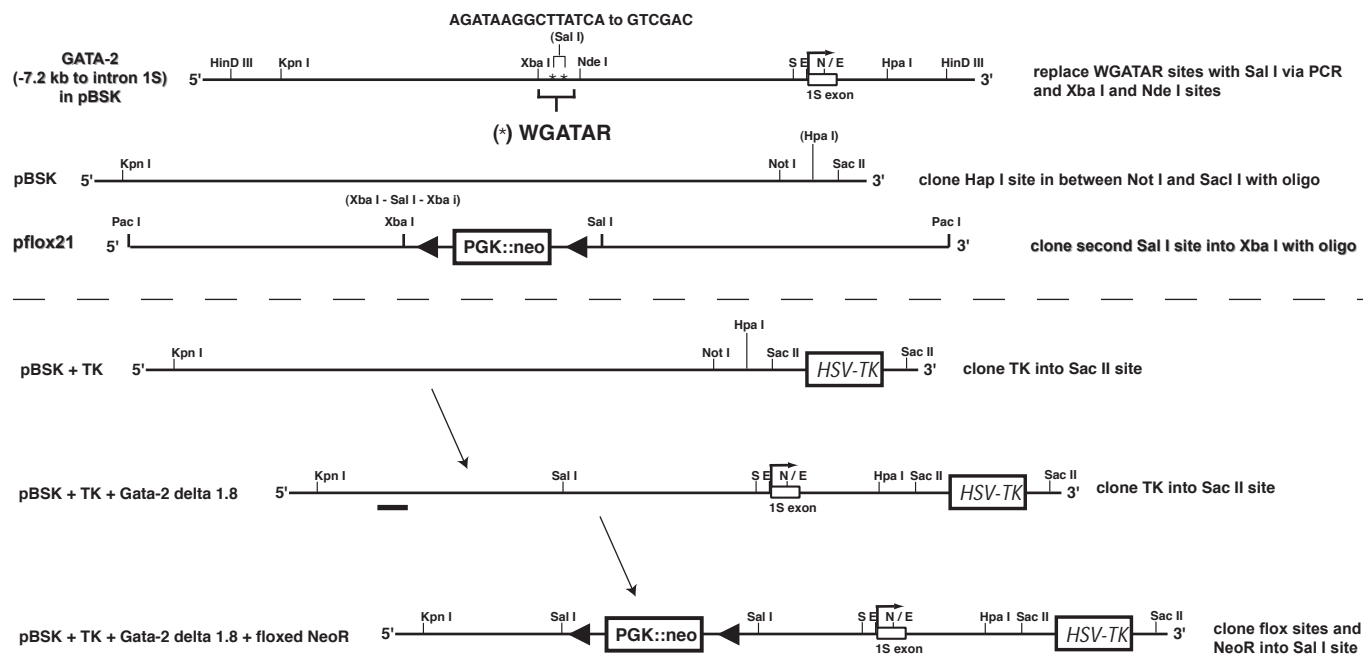

**B**

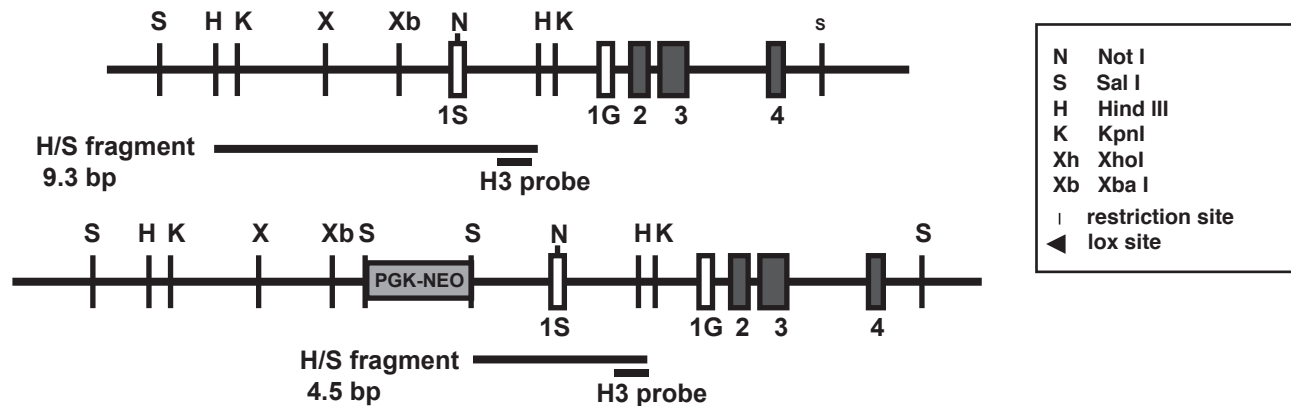

**C**

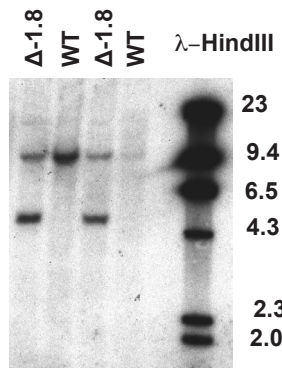

Supplement: Figure S1 — Δ-1.8 targeting construct generation. Graphical representation of the generation of the targeting construct to replace the palindromic GATA-binding site −1.8 kb upstream of the 1S transcriptional start site with a loxP-flanked PGK-neomycin cassette (A). Southern blot strategy outlining the HindIII/SalI digested fragment sizes for the wild-type and targeted alleles, and probe hybridization sites (B). Southern blot of Δ-1.8 germline mice and wild-type (WT) littermates from tail tip genomic DNA (C). (0.27 MB PDF) [file pgen.1001103.s001.pdf]

**A**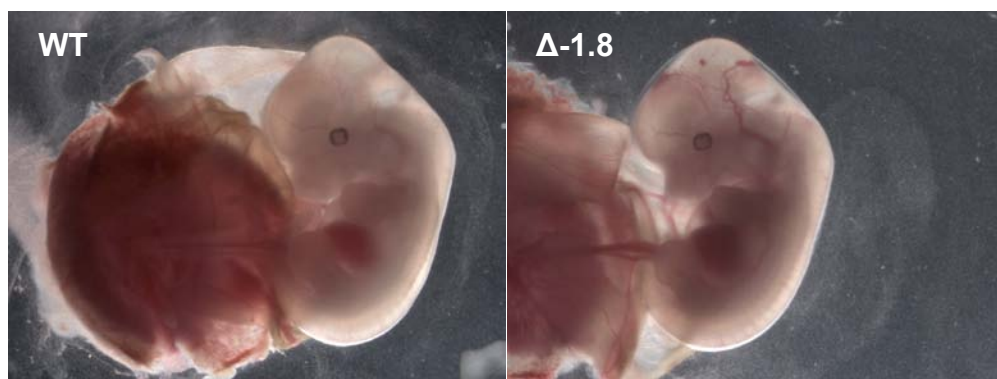**B****Peripheral Blood****Fetal Liver****WT**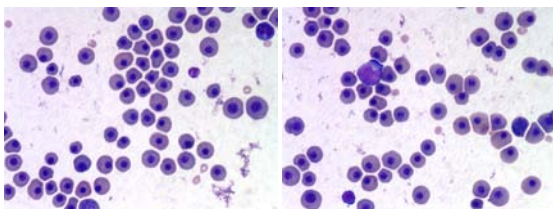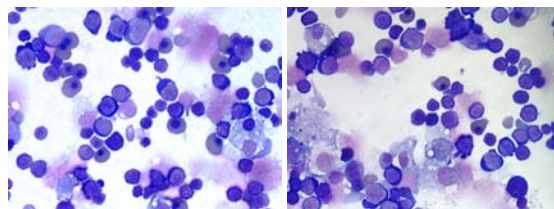**Δ-1.8**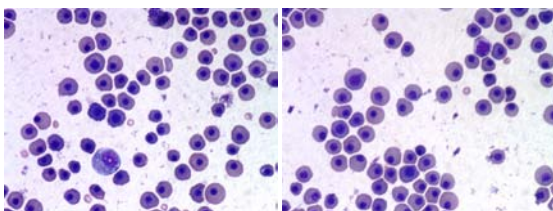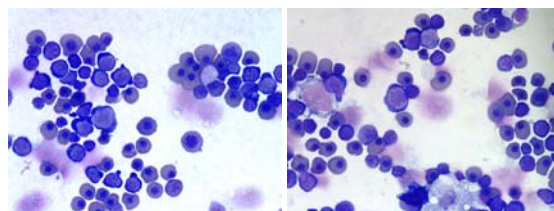**C**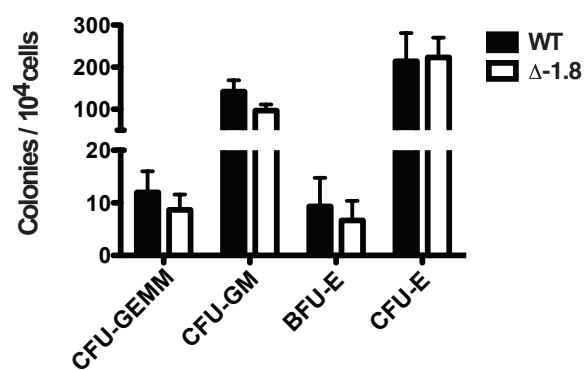**D**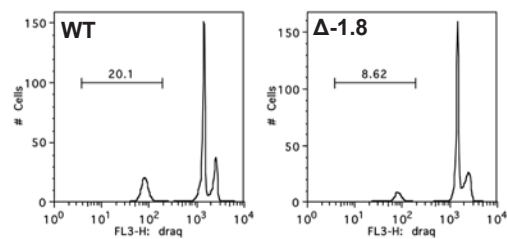

Supplement: Figure S2 — E12.5 hematopoiesis in Δ-1.8 mice. Representative E12.5 wildtype (WT) and Δ-1.8 embryos (A). Cytospins of embryonic peripheral blood and fetal liver cells from E12.5 WT and Δ-1.8 embryos (B). Number of CFU-GEMM, CFU-GM, BFU-E, and CFU-E colonies per 104 wild-type and Δ-1.8 E12.5 fetal liver cells (C). FACS histograms showing the proportion of enucleated cells from Stage IV erythroblasts within representative wild-type and Δ-1.8 fetal livers (D). (0.99 MB PDF) [file pgen.1001103.s002.pdf]

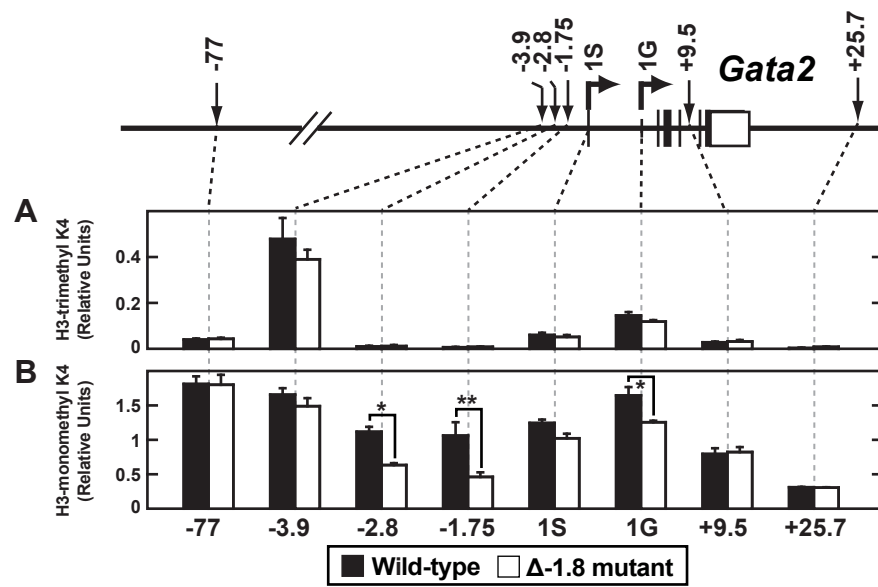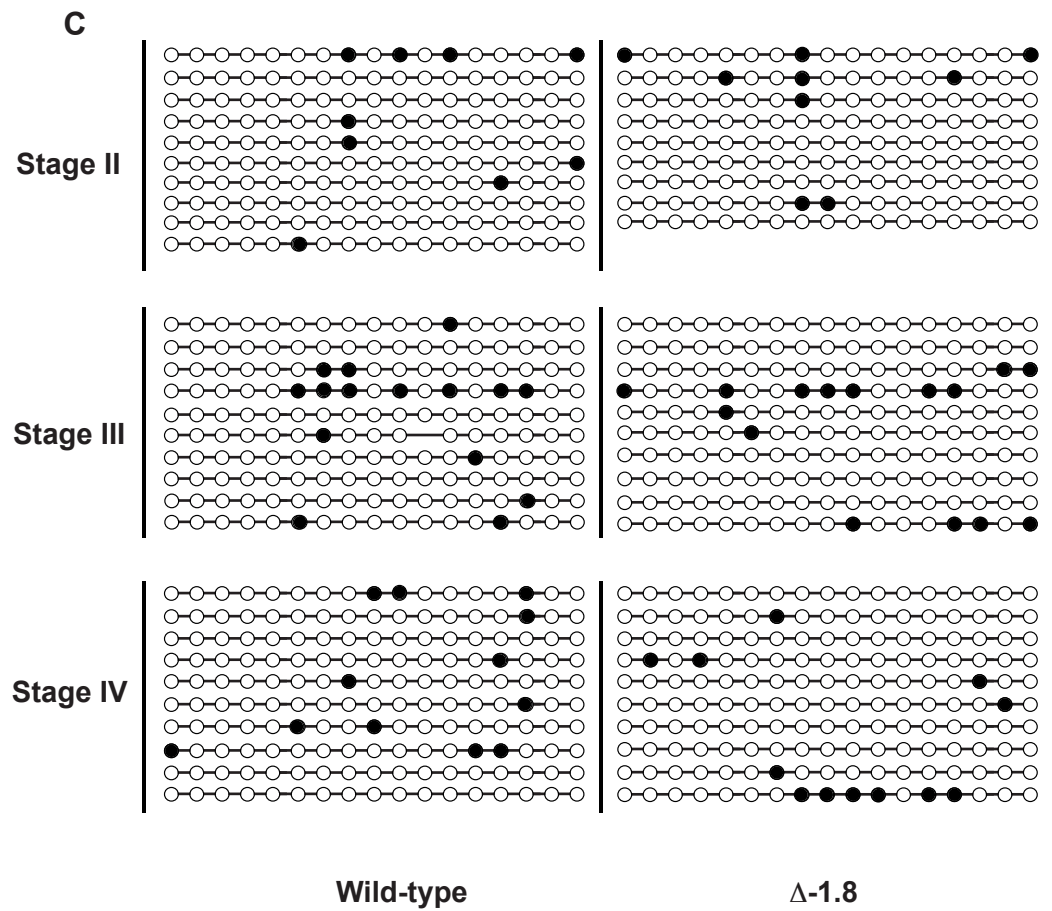

Supplement: Figure S3 — Loss of the −1.8 kb site leads to altered nucleoprotein architecture of the Gata2 locus. Quantitative ChIP analysis across the Gata2 locus using antibodies to trimethylH3K4 (A) and monomethylH3K4 (B) in whole fetal liver cells from wild-type and Δ-1.8 embryos at E14.5. Calculations were derived as above. Bisulfite sequencing of the 3′ region of the Gata2 1S promoter CpG island in WT and Δ-1.8 Stage II, Stage III, and Stage IV erythroid progenitors. Each line represents an individual sequenced clone; white circles denote unmethylated CpG dinucleotides, black circles denote methylated CpGs (C). (0.49 MB PDF) [file pgen.1001103.s003.pdf]
