# Supplementary material for: A Single cis Element Maintains Repression of the Key Developmental Regulator Gata2
Source: PLoS Genet. 2010 Sep 9;6(9):e1001103. doi: 10.1371/journal.pgen.1001103 (PMC2936534; doi:10.1371/journal.pgen.1001103)
Supplement: Text S1 — Supporting Materials and Methods. (0.05 MB DOC) [file pgen.1001103.s004.doc]

**Text S1**

| NAME | FORWARD | REVERSE |
| --- | --- | --- |
| Δ-1.8 Genotyping | cgaggccacctcattagaac | GCCATTTACTCTGGCACTTTC |
| Gata2 mRNA | GCAGAGAAGCAAGGCTCGC | CAGTTGACACACTCCCGGC |
| Gata2 1SE/E2 mRNA | GCCGCAGTCGGGCC | CTGCTCAGGCGCCACCT |
| Gata2 1GE/E2 mRNA | CCGCTGCGAGTGGCC | GCCCGGATGGTGCGA |
| Gata2 I2/E2 primary transcript | AGTGTCCTTCACATTCCCTCTGTT | TCTTGGGAACCAGGCGAA |
| Gata2 1SE/I2 primary transcript | GCGCCGCAGTCGGTAA | TTCCGGATGACCTGGGTTT |
| Gata2 1GE/I2 primary transcript | CGCCGCTGCGAGTGTAA | AACCTAGGAATGTCCACAAGCC |
| Gata1 mRNA | cagaaccggcctctcatcc | tagtgcattgggtgcctgc |
| Eklf mRNA | agactgtcttaccctccatcag | ggtcctccgatttcagactcac |
| Scl mRNA | cactaggcagtgggttctttg | ggtgtgaggaccatcagaaatct |
| Eraf mRNA | GATCTCCACAGGGATAAAGGAGTTT | CAGTCATGAACCACAATCACCAT |
| Alas2 mRNA | CCATCTTAAGGCAACCAAGGC | ACAGCATGAAAGGACAATGGC |
| Hba-a1 mRNA | GTGGATCCCGTCAACTTCAAG | CAAGGTCACCAGCAGGCAGT |
| Hbb-b1 mRNA | TTTAACGATGGCCTGAATCACTT | CAGCACAATCACGATCATATTGC |
| c-Kit mRNA | AGCAATGGCCTCACGAGTTCTA | CCAGGAAAAGTTTGGCAGGAT |
| c-Myb mRNA | TTACCAGGCACACAAGCGTCT | GAATTCCAGTGGTTCTTGATAGCA |
| Hbb-y mRNA | TGGCCTGTGGAGTAAGGTCAA | GAAGCAGAGGACAAGTTCCCA |
| Hbb-bh1 mRNA | TGGACAACCTCAAGGAGACC | ACCTCTGGGGTGAATTCCTT |
| Hba-x mRNA | TACCCCCAGACGAAGACCTA | CTTAACCGCATCCCCTACGG |
| Cpa-3 mRNA | AATTGCTCCTGTCCACTTTGAC | tgcctgcgatttcatctttcac |
| c-Mpl mRNA | aacccggtatgtgtgccag | agttcatgcctcaggaagtca |
| β-actin mRNA | aaggagattactgctctggctccta | actcatcgtactcctgcttgctgat |
| 18S RNA | CGCCGCTAGAGGTGAAATTCT | CGAACCTCCGACTTTCGTTCT |
| Gapdh | TGCCCCCATGTTTGTGATG | TGTGGTCATGAGCCCTTCC |

**Primer sequences.** The following primer sequences were used for genotyping and qPCR analysis.

**Colony-forming cell assay**

E12.5 fetal liver cells were plated at 2 x 10^5^ / ml in M3434 (Stem Cell Technologies, Vancouver, BC, Canada) as per manufacturer’s instructions and total colony-forming units (CFUs) as well as fraction of CFU-Granulocyte macrophage (CFU-GMs), erythroid burst-forming units (BFU-Es), and CFU- Granulocyte, erythroid, megakaryocyte, macrophage (CFU-GEMM) were enumerated at 10 days post plating. For CFU-E, 2 x 10^5^ cells / ml were plated in M3234 (Stem Cell Technologies, Vancouver, BC, Canada) as per manufacturer’s instructions and total colony-forming units and benzidine positive cells were enumerated at 3 days post plating.
